# Visualisation and biovolume quantification in the characterisation of biofilm formation in *Mycoplasma fermentans*

**DOI:** 10.1038/s41598-021-90455-5

**Published:** 2021-05-27

**Authors:** Ammar A. Awadh, Alison F. Kelly, Gary Forster-Wilkins, David Wertheim, Richard Giddens, Simon W. Gould, Mark D. Fielder

**Affiliations:** 1 School of Life Science Pharmacy, Chemistry Faculty of Science, Engineering and Computing, Kingston University London, Kingston, UK; 2 School of Computer Science and Mathematics, Chemistry Faculty of Science, Engineering and Computing, Kingston University London, Kingston, UK

**Keywords:** Biochemistry, Microbiology, Pathogenesis

## Abstract

The ability of mycoplasmas to persist on surfaces has been widely acknowledged, despite their fastidious nature. However, the organism’s capability to form a recognisable biofilm structure has been identified more recently. In the current study *Mycoplasma fermentans* was found to adhere to the glass surface forming highly differentiated biofilm structures. The volumes of biofilm microcolonies were quantified and observed to be greater at late growth stage than those at early growth stage. The channel diameters within biofilms were measured with Scanning Electron Microscopy images and found to be consistent with the size observed in Confocal Laser Scanning Microscope images. The combination of imaging methods with 3D visualisation provides key findings that aid understanding of the mycoplasma biofilm formation and true biofilm architecture. The observations reported here provide better understanding of the persistence of these minimalist pathogens in nature and clinical settings.

## Introduction

Mycoplasmas are known to possess a minimal genome and be extremely fastidious in terms of growth requirements and nutrition^1^. However, they are significant and successful medical and veterinary pathogens found to survive and persist in numerous niches including the mucosal surfaces of the respiratory and genitourinary tracts in both humans and animals^2–4^.

It has been reported in other organisms that biofilm formation aids bacterial persistence in a variety of situations and settings^5,6^. With this in mind, the mycoplasmal biofilm formation has been largely understudied despite its potential role in both medical and veterinary situations.

MacAuliffe et al.^7^ observed biofilm formation in species of veterinary mycoplasma whilst examining their survival and persistence under a number of environmental conditions. Simmons and Dybvig^8^ described the survival of *Mycoplasma pulmonis*, a rodent species, in a biofilm and the effects of *Vsa* short form protein on the organism’s resistance to complement and antibiotic therapy within a biofilm. In other studies researchers have examined the basic formation and architecture of the forming mycoplasmal biofilm^9–11^ where *M. pneumoniae* was examined for its capability to bind and form full biofilms on abiotic surfaces.

To fully appreciate the development and quantification of a biofilm in this genus, this work investigates a mycoplasma species of medical importance, *Mycoplasma fermentans*, by two microscopic techniques, scanning electron microscopy (SEM) and confocal laser scanning microscopy (CLSM), in order to visualise and quantify biofilm volume within the extracellular polymeric substances (EPS) which plays a crucial role in the increase of biofilm mass and viability, proposing a strong correlation between each other in the biofilm system^12^. This work describes the formation of distinct architectural structures within mycoplasma biofilms including towers and make measurements of this architecture and formal channel structures using novel image analysis technologies to reveal the components of the architecture. This paper details the confocal microscope imaging steps used to obtain high resolution 3D bacterial growth image datasets and detail measurements of the architecture revealed.

## Materials and methods

### Organisms used in the study

In this study *M. fermentans* from the Kingston University culture collection were utilised, briefly these were three clinical strains (MF1, M67195, M67910) provided by the late Dr David Pitcher and one type strain of *M. fermentans* (ATCC19989). All strains were obtained as freeze-dried cultures, and upon receipt, cultures were grown and sub-cultured in fresh Eatons broth medium and then stored at − 80 °C prior to use. Planktonic cells were grown using Eaton’s broth at 37 °C in a 5% CO_2_ atmosphere with no agitation for 24 h.

### Growth of biofilms with human *M. fermentans* strains

Biofilms were grown as described by McAuliffe et al.^7^. Briefly, sterile 22 mm^2^ glass coverslips (Sigma) were placed vertically into 50 ml sterile falcon tubes (Corning) containing 8 ml of pre-warmed Eaton’s medium. The tubes were then inoculated with a 1:100 dilution of a 20 h planktonic culture and left at 37 °C in a 5% CO_2_ atmosphere with no aeration for 3–7 days as required.

### Characterisation of biofilms using confocal laser scanning microscopy (CLSM)

This method was based upon Kornspan et al.^9^ with the following modifications.

Mycoplasma strains were grown on sterile glass coverslips (22 mm^2^) as described above for 3 and 7 days. Biofilm samples were grown in duplicate.

After incubating for 3 and 7 days, coverslips were washed twice with phosphate buffer saline (PBS) and fixed with 4% formaldehyde solution (Sigma, UK) in PBS for 10 min at room temperature. After fixation, coverslips were stained with propidium iodide/PBS solution for 15 min (at a ratio of 1:9). Lastly, the processed coverslips were washed twice with PBS, and mounted with solution containing 90% glycerol (Sigma, UK) and 10% PBS solution. These were imaged using a Leica TCS SP2 inverted confocal microscope (Leica GmbH, Germany). An oil immersion objective lens × 63 was used with a Numerical Aperture of 1.4. The CLSM was set to generate a series of 12-bit grey level resolution and using an Airy number of 1.0; the size of the axial slices was 1024 × 1024 pixels corresponding to 238 × 238 µm and these slices had a z-dimension spacing of 0.12 µm. The fluorescence of propidium iodide was excited using a 514-nm laser with the detection wavelength range of 539–629 nm. The image datasets were processed, visualised and quantified using Amira, version 5.4 software (Visualisation Sciences Group, USA). The first step involved removing noise by applying a median filter to each slice. The filtered images were then thresholded in order to define the microcolonies and produce 3D iso-surface visualisations.

The volume of attached biofilm cells was measured using Amira in 9 non-overlapping areas of the glass coverslip. The volume data of the microcolonies obtained with Amira were further analysed using Minitab version 16 (Minitab Inc., USA).

### Preparation and characterisation of mycoplasma samples by scanning electron microscopy (SEM)

All of *M. fermentans* strains were visualised using SEM and CLSM imaging application. Mycoplasma strains were grown on sterile glass coverslips as described above. Sample preparation for SEM analysis was carried out as described by Stadtlander^13^. Biofilm structures were examined using a Zeiss scanning electron microscope (Zeiss Evo 50).

The channel dimensions of the interstitial voids (water channels) within the biofilms were measured with software we developed using MATLAB (Version R2013b, The MathWorks Inc., USA).

### Ethical approval

This research raised no ethical concerns for consideration as no human or animal subjects included in the experimental work.

## Results

**Figure 1:**
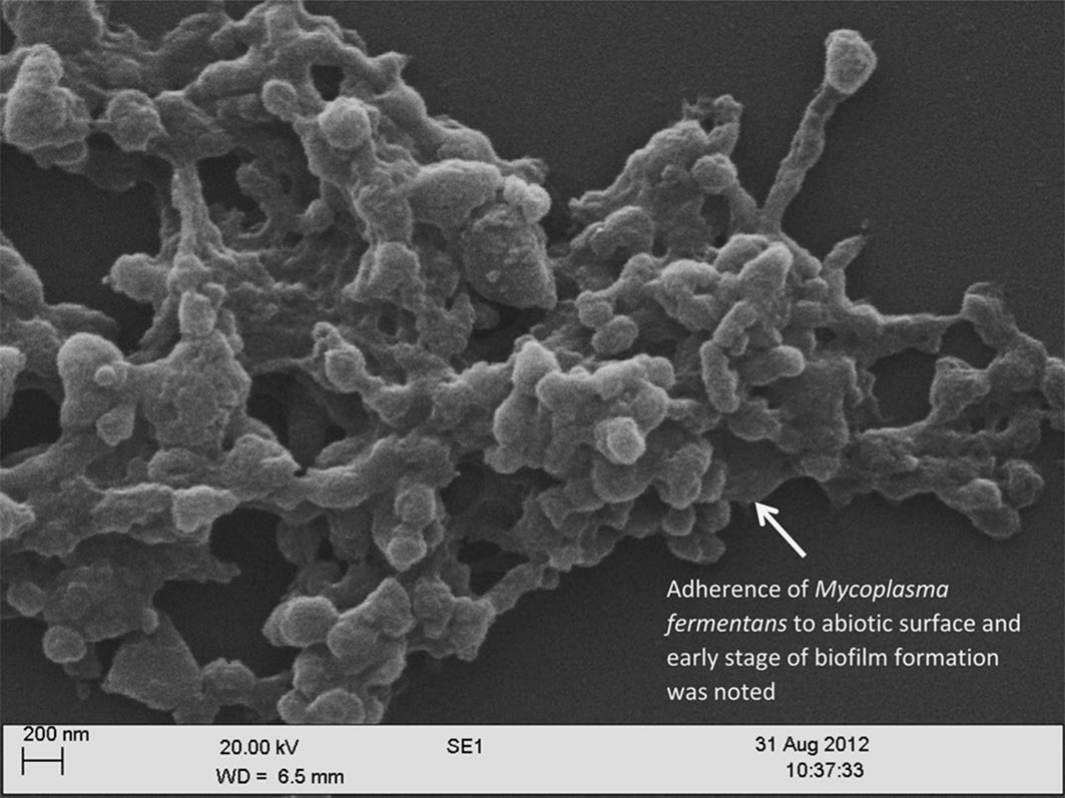
Representative scanning electron microscopy image of *Mycoplasma fermentans* ATCC19989 biofilm on glass coverslip illustrating an initial biofilm formation (arrow). This process was observed in all strains under examination.

**Figure 2:**
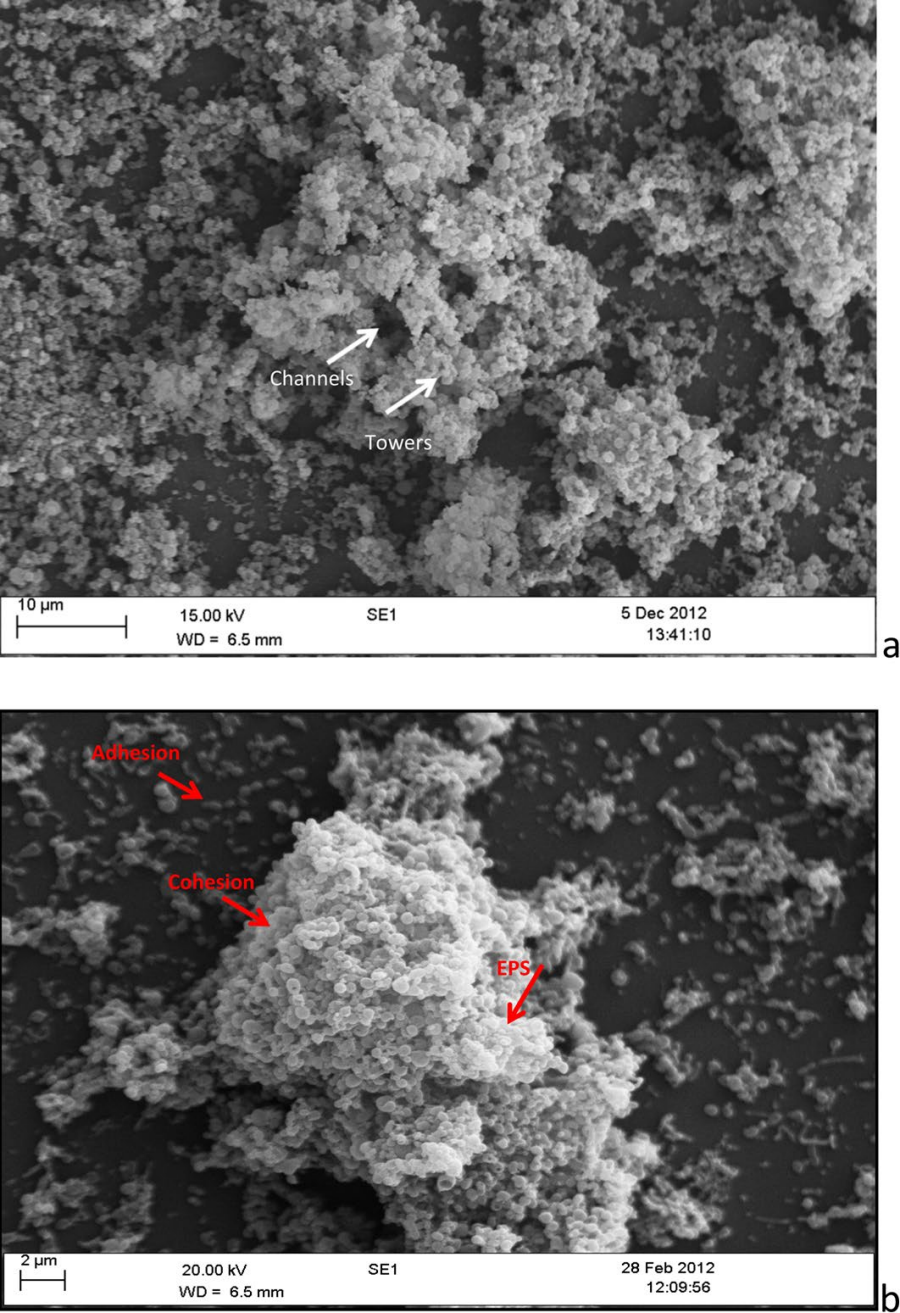
Scanning electron microscopy image of *Mycoplasma fermentans* ATCC19989 biofilm on glass coverslip illustrating complex biofilm architecture such as (**a**) channels and towers, (**b**) extracellular polymeric substances (EPS) embedding biofilm cells, adhesion and cohesion attachment of mycoplasma cell clusters (arrows).

**Figure 3:**
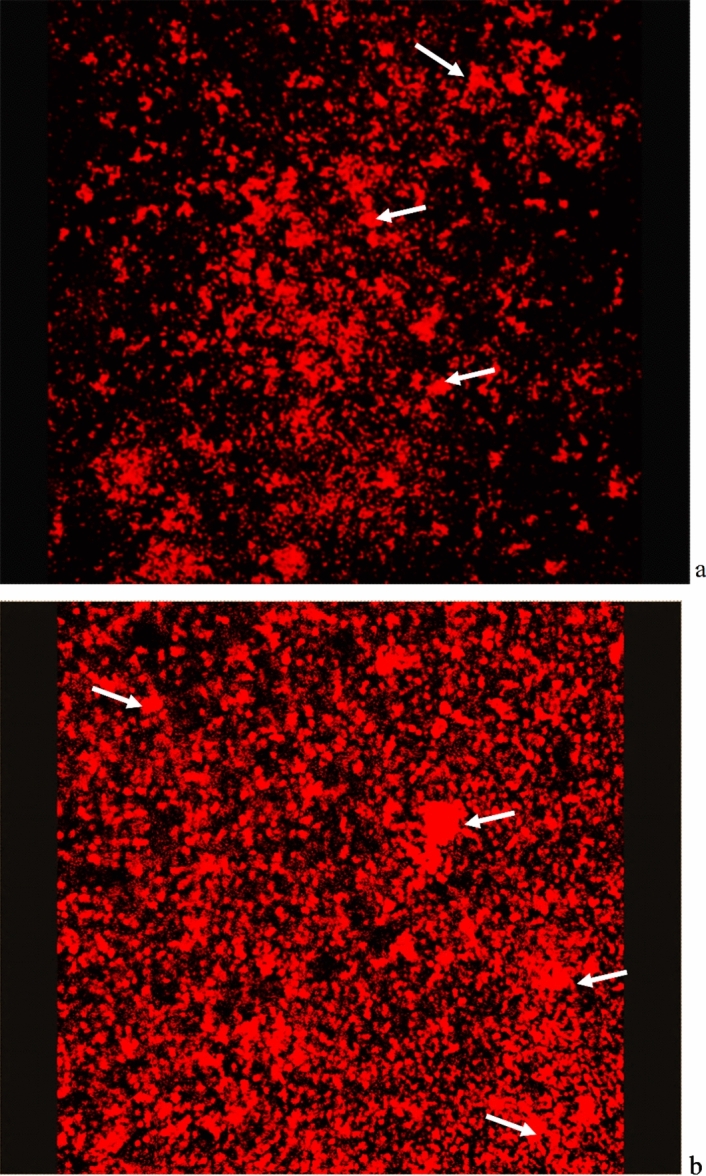
Fluorescent staining, propidium iodide, combined with confocal microscopy showing image of a biofilm formed by *Mycoplasma fermentans* ATCC19989 in Eaton’s broth medium, after 3 days (**a**) termed early growth stage and 7 days (**b**) termed late growth stage. The image was captured using a ×63 objective lens and the lateral dimension was 238 microns × 238 microns. The biofilm formed showed numerous tower structures (indicated by arrows).

All *M. fermentans* appeared to adhere to the abiotic surface and initial biofilm formation indicated by the arrow on Fig. 1, and as biofilm development continued more complex architectural structures including towers and channels formed (Fig. 2a). Imaging using SEM provided a clear indication of mycoplasmal biofilm architecture in 2D, such as tower structures and interstitial voids (water channels) as shown in Fig. 2a. SEM also revealed the fine structure in biofilm architecture, such as EPS that embeds the microbial cells and the method also showed the adhesion and cohesion attachment of mycoplasma cell clusters that embedded by the EPS (Fig. 2b). Confocal microscopy in combination with fluorescent staining showed microcolonies attached to the abiotic surface at early (3-day-old) and late (7-day-old) growth stage with biofilm architecture visible, as indicated by the arrows on Fig. 3 (Fig. 3a,b).

**Figure 4:**
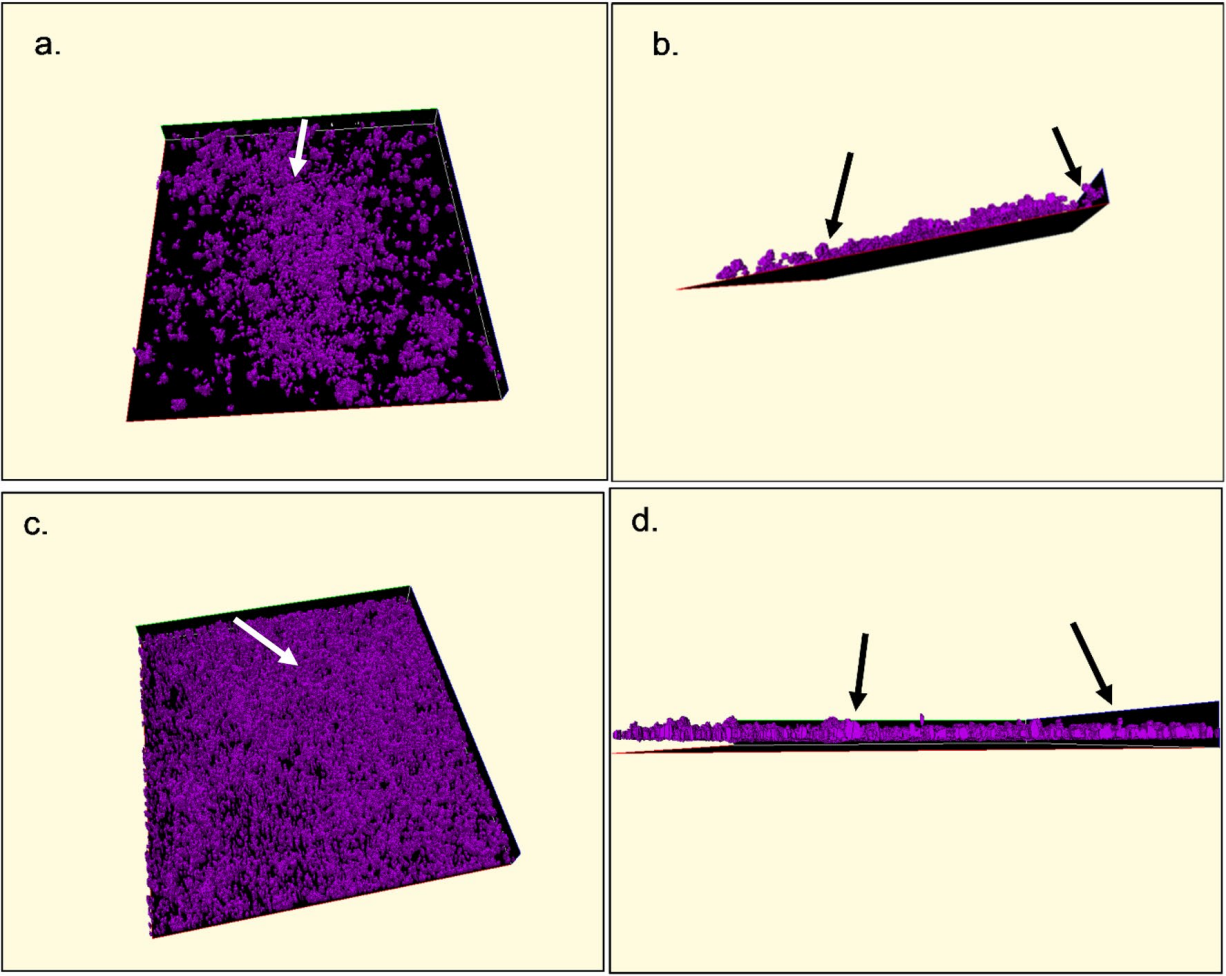
Three-dimensional visualisation confocal images of biofilm structure formed by *Mycoplasma fermentans* ATCC19989 after 3 days (**a**, **b**) and 7 days (**c**, **d**) termed late growth stage. Overhead views in (**a**, **c**) and side views for the early and late growth stage seen in (**b**, **d**). The lateral dimension of each confocal image was 238 microns × 238 microns. Arrows indicate the examples of tower structures in both early and late stage biofilm growth (Amira ver 5.4 https://www.thermofisher.com/uk/en/home/industrial/electron-microscopy/electron-microscopy-instruments-workflow-solutions/3d-visualization-analysis-software.html, https://www.vsg3d.com).

The confocal laser scanning microscopy provided cross-sections through biofilm structures used to create 3-D visualisations of biofilm architecture at early and late stage growth (Fig. 4a–d). As a result, the data could be quantified to determine the volume of biofilm cells adhered to the glass substratum. The biovolume of *M. fermentans* strains biofilms were determined by calculating the median volume of at least five adhesion points on the surface, the median was calculated and the data interval indicated the increasing rate in biofilm volume over time (Table 1).

**Table 1:** The quantification analysis of biofilm volume in *Mycoplasma fermentans* strains growth at 3 and 7 days by calculating the median for each replicate.

*Mycoplasma fermentans* biofilm samples	Median of biofilm cells growth at 3 and 7 days (µm^3^) × 10^3^	
	Median 3 days	Median 7 days
***M. fermentans* ATCC19989**		
Replicate 1	76	97
Replicate 2	27	106
***M. fermentans* (M67910)**		
Replicate 1	4.9	5.8
Replicate 2	1.9	46
***M. fermentans* (MF1)**		
Replicate 1	7.7	40
Replicate 2	40	21
***M. fermentans* (M67195)**		
Replicate 1	1.9	2.0
Replicate 2	2.0	3.9

There were clear differences between the volumes calculated for each sample, however in all but one case there was an increase in volume by 7 days. As a result of the observed variability the media value is used and the increase in the median value over time is recorded, exemplar data shown in “S1”. *M. fermentans* (M67195) and *M. fermentans* (M67910) showed the least growth at both 3 days and 7 days, whilst the highest growth was seen with *M. fermentans* ATCC19989, as assessed by biofilm volume. With reference to Fig. 4a–d, it can be seen that changes in the biovolume are mimicked by alterations in the biofilm structure and architecture. Where the biofilm coverage and apparent biofilm height has increased from day 3 (Fig. 4a,b) and similar planes of vision of the same strain on day 7 (Fig. 4c,d).

**Figure 5:**
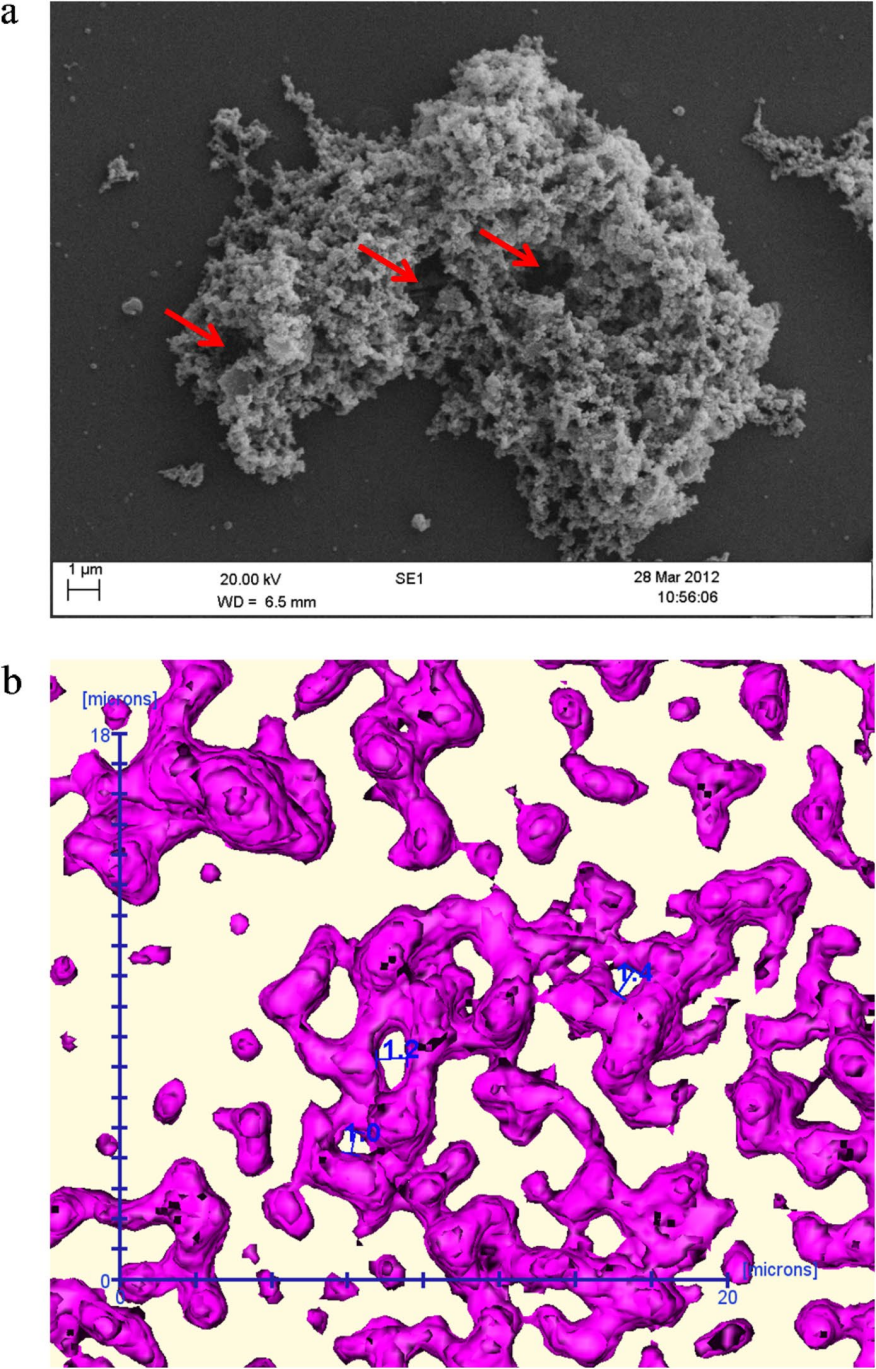
(**a**) Scanning electron microscopy image of *Mycoplasma fermentans* ATCC19989 determining channel diameter sizes compared to (**b**) that with confocal laser scanning microscopy (MATLAB ver R2013B, https://uk.mathworks.com/) (Amira ver 5.4 https://www.thermofisher.com/uk/en/home/industrial/electron-microscopy/electron-microscopy-instruments-workflow-solutions/3d-visualization-analysis-software.html, https://www.vsg3d.com).

As shown in Fig. 5a,b demonstrate examples of channels within towers viewed by SEM in comparison with water channel measurements obtained from confocal microscopy. The channel diameters seen with CLSM are about 1 µm (shown in blue text) and appear to be consistent with the larger channels seen on SEM imaging and hence likely not to be an artefact.

## Discussion

*Mycoplasma fermentans* is a pathogen implicated in several human diseases including rheumatoid arthritis (RA) and conditions such as bacteraemia^14,15^. Horowitz et al.^16^ detected *M. fermentans* DNA in synovial fluids from 17.6% of patients with RA whilst Gilroy et al.^17^ detected *M. fermentans* DNA in synovial fluid of 17% of patients with RA and in 21% of patients with seronegative arthritis. In addition, Gil et al.^15^ found evidence of *M. fermentans* involvement in bacteraemia in a high percentage of RA patients in Mexico.

Microbial biofilms and the matrix components have previously been investigated by means CLSM, which has been considered a valuable method to view biofilm development^18^. Electron microscopy also provides high resolution magnification that offers further insight into the ultrastructure of biofilms. Several EM techniques have previously been employed in order to investigate biofilm structures including scanning electron microscopy (SEM)^19,20^. This study combines both CLSM and SEM techniques for the first time the fine to elucidate and quantify the architecture of mycoplasmal biofilms.

The images of biofilm architecture analysed in the current study viewed using SEM revealed structures in human mycoplasmas biofilm, including tower structures, channels and densely packed cells in the towers as previously hypothesised by Simmons and Dybvig^8^ and the current work indicated that biofilm formation by human mycoplasma isolates varied both qualitatively and quantitatively.

It was possible to obtain a high-resolution image of *M. fermentans* aggregation on the abiotic surface using SEM as well as visualisation of complex biofilm structures (Fig. 1). A number of reports on biofilm formation agree that biofilm cells are embedded in a matrix of extra cellular polysaccharide substances (EPS)^21^, which are composed of polysaccharides and a wide variety of components, such as glycoprotein, glycolipids, and in some cases, surprising amounts of extracellular DNA (e-DNA)^22^. When bacteria attach to a surface (biotic or abiotic), they are often found to be encased in a thin slime layer known as EPS. The EPS is comprised of a set of macromolecules, such as lipoglycans, glycolipids and a polysaccharide capsule^22^. This could be observed in Fig. 1, where the attached cells on a surface found encased in a slime thin layer (EPS). This attachment then lead onto the formation of mature biofilms that can be observed in Figs. 2 and 3. Moreover, EPS often has a defined macromolecular “honeycomb” structure^22^ and this could be seen in Fig. 2a,b. This honeycomb structure was also noted in the EPS of the biofilms of *M. pneumoniae* by Kornspan et al.^9^, the similarity of the observations in the two different studies suggest that these structures are likely to be the EPS in the biofilm in the *Mycoplasma* species in the current study.

Previously, it was determined that biofilms of veterinary mycoplasmas were found to form highly differentiated structures with channels and stacks similar to those found in *Pseudomonas aeruginosa* and *Staphylococcus epidermidis*^7,23^. In this work, human mycoplasma biofilm structures were visualised and characterised, and the data suggests that the biofilm structures observed could be similar in all microbial biofilms potentially including other human mycoplasmas species.

Using CLSM it has been possible to visualise general biofilm structures including tower structures as previously described^8,9,11^. Here the technique was expanded to measure and quantify these structures as well as water channels within biofilms. The obtained images of biofilm structures (Fig. 4a–d) showed the general biofilm structure where the bright heavy stained cells were considered as towers and the regions that did not contain cells were recognised as black background.

Additionally, CLSM can be used to scan biofilm structures into cross-sections^9^ and these were used to depict three-dimensional images (Fig. 4a–d). The use of CLSM allowed for on-invasive inspection and subsequent computer reconstruction of a mature biofilm without appreciable distortion of the architecture, creating an enhanced conceptual image of microbial biofilm architecture as it exists in situ^23^. Furthermore, this method allowed biofilm architecture to be viewed from different angles and therefore identifying general structures such as towers (Fig. 4a–d). Thus, the use of CLSM and computerised image analysis could reveal a more complex view of biofilm morphology and architecture^24^.

It is tempting to visualise microbial biofilms as a mass of micro-organisms uniformly distributed throughout a polysaccharide matrix overlying a surface^23^. In this way, the microbial cells multiply within the embedded exopolysaccharide matrix giving rise to microcolonies^25^. Biofilms are composed of approximately 10–25% microbial cells and 75–90% EPS by volume^26^. The view proposed by^12^ stated that EPS plays a critical role in the biofilm system, especially in mass and viability, where increasing volume of biofilm was correlated with EPS. CLSM is a tool that could be useful to detect and visualise biofilm matrix components^18^, which in turn could provide detailed quantitative characterisation of internal microstructures^27^.

The current work describes the volume of human mycolasmal biofilm architecture. The mycoplasmal communities were measured after two different growth periods, 3 and 7 days respectively, to take into consideration constantly changing biofilm architecture in both space and time^23^. The volumes of biofilm after each time point were quantified by measuring a number of randomly chosen areas on the surface (Table 1, “S1”). The aim of obtaining various measurements was to rule out experimental bias and to determine if there was a difference in volume or size of biofilms. The results showed that the volumes of biofilms were greater at late growth stage (7 days of growth) than those at early growth stage (3 days of growth) (Table 1, “S1”) and this may suggest that the volume of microcolonies increase with time as the biofilms get older. This finding, along with the observation stated that low-density regions extended throughout the entire depth of the EPS matrix, may be indicative of some degree of developmental structural organisation. It may be speculated that these thin biofilms were in some way immature and that as they continue to grow and thicken and thus potentially increase the volumes of biofilms.

The current work also identified channel structures “water channels” within a developed biofilm (Fig. 2a). An earlier study^28^ hypothesised that certain criteria considered as characteristics of biofilms, including a thin base layer, ranging from a patchy monolayer of cells to a film several layers thick containing water channels, which may allow the diffusion of nutrients, oxygen, and even antimicrobial agents^29^. Therefore, the combination of both SEM and CLSM was applied in order to determine and quantify channel structures by measuring their diameter (Fig. 5). This is the first time that these architectural structures have been visualised and quantified in terms of biovolume of cells embedded within the EPS. The microscopic image in Fig. 5 provide a realistic, high resolution image of the observed biofilm architecture, especially ultra-structures including water channels that could be targeted for further studies toward understanding the biofilm formation process in these organisms.

The channels within biofilms were identified and measured and diameters associated with SEM images were found to be approximate to those associated with CLSM micrographs (Fig. 5). This infers that two-dimensional biofilm architecture could be applicable to those in three-dimensional biofilms. Moreover, these findings suggest that the visualised channels might correspond to those previously hypothesised in biofilm architecture^8,30^. Using both CLSM and SEM these channels were found in mature biofilms as open funnels surrounded by microcolonies (Fig. 5). Using a biofilm liquid flow model^31^, water flow was demonstrated and tracked inside a bacterial biofilm through channels (0.28 µm diameter) where it was estimated that the average flow velocity of the water through the biofilm was 6.6 cm/s and observed that the flow velocity would become faster (up to 10–20 cm/s) as channel diameter size became bigger. In the current study, water channels sizes in *M. fermentans* strains tested were found to be larger, ranging approximately from 0.2 to 2.07 µm in diameter. Increased channel size has also has been found to affect biofilm formation, providing an effective means of exchanging nutrient and metabolites with bulk aqueous phase, enhancing nutrient availability as well as removal of potentially toxic metabolites^32^. Therefore, the discovery of water channels in mycoplasma biofilms, suggests that fluid could flow inside biofilm and has important consequences for understanding fundamental biofilm processes.

This combination of visualisation methods has been beneficial in the quantification of the structure of biofilms in human mycoplasmas, providing a better description and understanding of mycoplasmal biofilm systems including which structures may affect their development. In the current study, the use of CLSM has facilitated the elucidation of the fine detail of mycoplasmal biofilm architecture and so allowed characterisation of the architecture of human mycoplasma biofilms.

This is the first report of using two independent microscopy techniques to visualise, measure and quantify biofilm architecture of human mycoplasma biofilms where details of the confocal imaging steps are provided on how the high-resolution 3D were obtained. This is an important observation allowing a greater understanding of how mycoplasma biofilms develop and function with the elucidation of defined structures and a measurable biovolume and add to the information provided in previous studies^10,11^. Similar observations have been made in previous studies^11^. However, aspects of the confocal microscope image acquisition methods are not clear in the paper by Simmons et al.^11^. In the current paper the 3D visualisations clearly have higher resolution as a result of the approaches employed. Furthermore, the high-resolution confocal microscopy methodology employed has enabled detailed visualisation of the structure of the biofilms. These observations include the identification of micro-channels in biofilm towers with diameters of the order of 1 µm and these were consistent with those observed on SEM imaging. The cavities/channels in the Simmons et al.^11^ appear to be larger of the order of 5 µm and manly shown in 2D. The current work utilised a specialised high resolution CLSM image acquisition approach as was able to detect and recognise the existence of micro-channels as small as 1 µm in diameter as well as enabling detailed 3D visualisation and assessment of biofilms. These novel findings are important in adding to the understanding of how these important ubiquitous minimalist pathogens are able to survive and persist in the host and the surrounding environment and may inform how control of these pathogens may be directed.

## Supplementary Information

Supplementary Information.

## References

[1] Staats CC, Boldo J, Broetto L, Vainstein M, Schrank A (2007). Comparative genome analysis of proteases, oligopeptide uptake and secretion systems in *Mycoplasma* spp. Genet. Mol. Biol..

[2] Afshar B, Nicholas RA, Pitcher D, Fielder MD, Miles RJ (2009). Biochemical and genetic variation in *Mycoplasma fermentans* strains from cell line, human and animal sources. J. Appl. Microbiol..

[3] Cassell GH, Clyde WA, Davis JK, Razin S, Barile MF (1985). Mycoplamal respiratory infections. The Mycoplasmas: Mycoplasma Pathogenicity.

[4] Roachford O, Nelson KE, Mohapatra BR (2019). Virulence and molecular adaptation of human urogenital mycoplasmas: A review. Biotechnol. Biotechnol. Equip..

[5] Sharma D, Misba L, Khan AU (2019). Antibiotics versus biofilm: An emerging battleground in microbial communities. Antimicrob. Resist. Infect. Control.

[6] Yan J, Bassler LB (2019). Surviving as a community: Antibiotic tolerance and persistence in bacterial biofilms. Cell Host Microbe.

[7] McAuliffe L, Ellis RJ, Miles K, Ayling RD, Nicholas RAJ (2006). Biofilm formation by mycoplasma species and its role in environmental persistence and survival. Microbiology.

[8] Simmons WL, Dybvig K (2007). How some mycoplasmas evade host immune responses. Microbe.

[9] Kornspan JD, Tarish M, Rottem S (2011). Adhesion and biofilm formation of *Mycoplasma pneumoniae* on abiotic surfaces. Arch Microbiol..

[10] Feng M, Schaff AC, Balish MF (2020). *Mycoplasma pneumoniae* biofilms grown in vitro: Traits associated with persistence and cytotoxicity. Microbiology.

[11] Simmons WL, Daubenspeck JM, Osborne JD, Balish MF, Waites KB, Dybvig K (2013). Type 1 and Type 2 strains of *Mycoplasma pneumoniae* form different biofilms. Microbiology.

[12] Joubert LM, Wolfaardt GM, Botha A (2006). Microbial exopolymers link predator and prey in a biofilm system. Microbial Ecol..

[13] Stadtlander CTKH, Mendez-Vilas A, Diaz J (2007). Scanning electron microscopy and transmission electron microscopy of mollicutes: Challenges and opportunities. Modern Research and Educational Topics in Microscopy.

[14] Kawahito Y, Ichinose S, Sano H, Tsubouchi Y, Kohno M, Yoshikawa T, Tokunaga D, Hojo T, Harasawa R, Nakano T, Matsuda K (2008). *Mycoplasma fermentans* glycolipid-antigen as a pathogen of rheumatoid arthritis. Biochem. Biophys. Res. Commun..

[15] Gil C, Rivera A, Bañuelos D, Salinas S, García-Latorre E, Cedillo L (2009). Presence of *Mycoplasma fermentans* in the bloodstream of Mexican patients with rheumatoid arthritis and IgM and IgG antibodies against whole microorganism. BMC Musculoskelet. Disord..

[16] Horowitz S, Evinson B, Borer A, Horowitz J (2000). *Mycoplasma fermentans* in rheumatoid arthritis and other inflammatory arthritides. J Rheumatol..

[17] Gilroy CB, Keat A, Taylor-Robinson D (2001). The prevalence of *Mycoplasma fermentans* in patients with inflammatory arthritides. Rheumatology (Oxford).

[18] Alhede M, Qvortrup K, Liebrechts R, Hoiby N, Givskov M, Bjarnsholt T (2012). Combination of microscopic techniques reveals a comprehensive visual impression of biofilm structure and composition. FEMS Immunol. Med. Microbiol..

[19] Priester JH, Horst AM, Van de Werfhorst LC, Saleta JL, Mertes LA, Holden PA (2007). Enhanced visualisation of microbial biofilms by staining and environmental scanning electron microscopy. J. Microbiol. Methods..

[20] Sangetha S, Zuraini Z, Suryani S, Sasidharan S (2009). *In situ* TEM and SEM studies on the antimicrobial activity and prevention of *Candida albicans* biofilm by *Cassia spectabilis* extract. Micron.

[21] Simmons WL, Dybvig K (2009). Mycoplasma biofilms ex vivo and in vivo. FEMS Microbiol. Lett..

[22] Flemming H, Neu TR, Wozniak DJ (2007). The EPS matrix: The “House of Biofilm Cells”. Am. Soc. Microbiol..

[23] Dune WM (2002). Bacterial adhesion: Seen any good biofilms lately?. Clin. Microbiol. Rev..

[24] Zhao W, Tan J, Qiu L (2005). Improvement of confocal microscope performance by shaped annular beam and heterodyne confocal techniques. Optik Optics..

[25] El Abed S, Ibnsouda SK, Latrache H, Hamadi F, Kazmiruk V (2012). Scanning electron microscopy (SEM) and environmental SEM: Suitable tools for study of adhesion stage and biofilm formation. Scanning Electron Microscopy.

[26] Garret TR, Bhakoo M, Zhang Z (2008). Bacterial adhesion and biofilms on surfaces. Prog. Natl. Sci..

[27] Wang Y, Chang H-I, Wertheim DF, Jones AS, Jackson C, Coombes AGA (2007). Characterisation of the macroporosity of polycaprolactone-based biocomposites and release kinetics for drug delivery. Biomaterials.

[28] Stoodley P, Boyle JD, Dodds I, Lappin-Scott HM, Wimpenny JWT, Gilbert PS, Lappin-Scott HM, Jones M (1997). Consensus model of biofilm structure. Biofilms: Community Interactions and Control.

[29] Donlan RM (2002). Biofilms: Microbial life on surfaces. Emerg. Inf. Dis..

[30] Behlau I, Gilmore MS (2008). Microbial biofilms in ophthalmology and infectious disease. Arch Ophthalmol..

[31] Stoodley P, De Beer D, Lewandowski Z (1994). Liquid flow in biofilm system. App. Environ. Microbiol..

[32] Kokare CR, Chakraborty S, Khopade AN, Mahadik KR (2009). Biofilm: Importance and applications. Indian J. Biotech..

