# Supplementary material for: Visualisation and biovolume quantification in the characterisation of biofilm formation in Mycoplasma fermentans
Source: Sci Rep. 2021 May 27;11:11259. doi: 10.1038/s41598-021-90455-5 (PMC8160185; doi:10.1038/s41598-021-90455-5)
Supplement: Supplementary file 1 — Supplementary Information. [file 41598_2021_90455_MOESM1_ESM.docx]

Supplementary information:

Visualisation, Biovolume Quantification and Characterisation of Biofilm Formation in *Mycoplasma fermentans*.

Ammar A. Awadh, Alison F. Kelly, Gary Forster-Wilkins, David Wertheim, Richard Giddens, Simon W. Gould, Mark D. Fielder

S1

Volumes of biofilm cells in *Mycoplasma fermentans* (MFType) from confocal

microscopy data. The median is highlighted.

| *Mycoplasma fermentans*  biofilm samples | Volume of biofilm cells growth at 3 and 7 days | |
| --- | --- | --- |
| ***M. fermentans (*MFType)** | **3 days** | **7 days** |
| Sample 1 | 63,541.36 | 110,967.12 |
|  | 68,120.91 | 105,137.73 |
|  | 100,740.66 | 96,893.80 |
|  | 262,103.44 | 91,032.73 |
|  | 75,577.40 | 78,577.02 |
|  | | |
| Sample 2 | 100,740.23 | 59,781.17 |
|  | 27,128.64 | 106,240.07 |
|  | 21,787.42 | 108,938.20 |
|  | 40,789.80 | 204,418.44 |
|  | 25,944.76 | 104,398.41 |

Scatter chart showing the median volume of biofilm cells after 3 days and 7 days of growth (•) represents biofilm samples () represents the median of two biofilm samples.
